# Temperature Control of Quartz-Glass Melting Areas in Laser Additive Manufacturing

**DOI:** 10.3390/mi16010029

**Published:** 2024-12-28

**Authors:** Jing Chen, Zeping Lv, Xuanjia Zhang, Tao Xu, Yuntao Cheng

**Affiliations:** 1 Lightweight Optics and Advanced Materials Technology Center, Institute of Optics and Electronics, Chinese Academy of Sciences, Chengdu 610209, China; c19950905250@163.com (J.C.); lvzeping22@mails.ucas.ac.cn (Z.L.);; 2 University of Chinese Academy of Sciences, Beijing 100049, China

**Keywords:** quartz glass, additive manufacturing, temperature control, coaxial wire feeding, direct energy deposition

## Abstract

Direct energy deposition is an additive technology that can quickly manufacture irregularly shaped quartz-glass devices. Based on this technology and coaxial laser/wire feeding, open-loop tests were conducted under different process parameters. A closed-loop temperature control system was designed and built for the molten pool temperature in quartz-glass additive manufacturing. It was based on a PID (proportional–integral–derivative) control algorithm for adjusting laser power. Changes in the macroscopic morphology, microstructure, and other qualities of the final additive result before and after the temperature control of the quartz glass were examined. Relative to constant laser powers of 120 W and 140 W, the temperature control of the multi-pass single-layer lateral additives produced dense surface microstructures of the additively produced quartz glass, and the molding quality was better.

## 1. Introduction

Quartz glass has excellent physical and chemical properties, such as high light transmittance, good heat resistance, and a low thermal expansion coefficient. It can be used in artificial kidneys in medical components, in semiconductor machine components, and in large parts for the construction industry [1]. Hence, there is considerable demand for it. At present, most of these large or complex quartz-glass products adopt subtractive methods, such as grinding and water cutting. These methods have low material utilization, complex processes, and high costs. Direct energy deposition (DED) technology is a direct additive technology that can quickly melt and then deposit quartz glass through an energy source. In theory, it can improve the utilization rate of materials and meet the need for quartz glass with multiple sizes, high yield rates, and complex shapes. However, because of its high melting point, brittleness, and high hardness, additive manufacturing technologies for quartz glass have developed slowly relative to those for metals, ceramics, and polymers [2].

Recently, quartz-glass additive technologies have made significant progress. According to different feeding methods and laser angles, DED can be divided into two types: coaxial and side-axis wire feeding. Luo et al. [3,4] used a CO_2_ laser in a DED printing system with side-axis wire feeding to additively manufacture fused quartz and print continuous single rails, single walls, cylinders, and cubes. Witzendorf et al. [1] used side-axis wire feeding to adjust the glass-fiber feed rate, laser power, and platform speed parameters to obtain repeatable and stable parameters for quartz-glass additive manufacturing. Grabe et al. [5] designed a coaxial wire feeding device for laser-fused silica-glass deposition and used numerical simulations to describe the temperature distribution of the glass fiber during heating. When coaxial and side-axis feeding are compared, coaxial feeding has the advantages of simplified system operation and the additive manufacturing of free-shaped objects. Lang Ming et al. [6] also prepared quartz glass by coaxial powder feeding. The maximum mechanical strength and thermal properties of the obtained quartz-glass components were close to those of quartz-glass components prepared by traditional methods.

Due to the high viscosity of quartz glass, the lack of a fixed melting point, and the fact that its thermal conductivity changes with temperature, defects such as bubbles and pores may appear in the final additively manufactured quartz glass, resulting in inconsistent and uneven forming quality [7,8]. Therefore, the temperature control method is selected to balance the energy in the additive manufacturing process, reduce defects caused by temperature changes, ensure the quality of the formed parts, and enhance the anti-interference ability of the processing process. In additive manufacturing that uses lasers as energy sources, there have been few studies on the temperature monitoring and control of quartz-glass materials, which are more common for metal and alloy materials. For these materials, direct-contact temperature measurement was usually performed by thermocouples [9] and infrared cameras [10], or pyrometers [11,12] for indirect temperature measurement. Among them, thermocouple temperature measurement has a low cost but low accuracy; infrared cameras can quickly measure temperature distribution over a large area, with high accuracy but a high price; and pyrometers are suitable for single-point ultrahigh-temperature rapid measurement, are portable, and have a fast response, but their accuracy is easily affected by surface emissivity. In the laser additive process, linear control [12,13], such as simple proportional (P) control, and nonlinear control methods [12,14], such as sliding mode control and adaptive control, were used to achieve temperature control. Linear control theory and applications are well developed, simple in design and easy to implement, and are often used in industrial scenarios such as temperature control and pressure control.

In order to achieve the rapid additive manufacturing of quartz glass and solve the problem of defects in quartz glass during laser additive manufacturing, we introduced a temperature control strategy based on DED technology combined with a coaxial wire feeding method. Initially, with consideration of the high temperature of the quartz-glass melting area and the need for fast real-time measurement, a pyrometer was selected as the temperature-measuring instrument. On this basis, an open-loop experiment was conducted to obtain a system model of power and temperature. A PID (proportional–integral–derivative) simulation was performed, in which the PID parameters were corrected via experiments. Then, the relationship between temperature and the quality of the results from a single-pass single-layer quartz-glass additive process was analyzed. Comparisons between open-loop and closed-loop temperature control in single-pass single-layer and multi-pass single-layer cases were also performed. Finally, via closed-loop temperature control, quartz glass with a complete surface structure and good molding quality could be obtained. This shows that the introduction of closed-loop temperature control is a feasible and practical technical solution, which can improve the surface morphology and material properties of additively produced quartz glass and lay the foundation for the further rapid realization of high-precision, high-quality, complex, three-dimensional-structure quartz glass.

## 2. Materials and Methods

### 2.1. Experimental Setup

**Figure 1:**
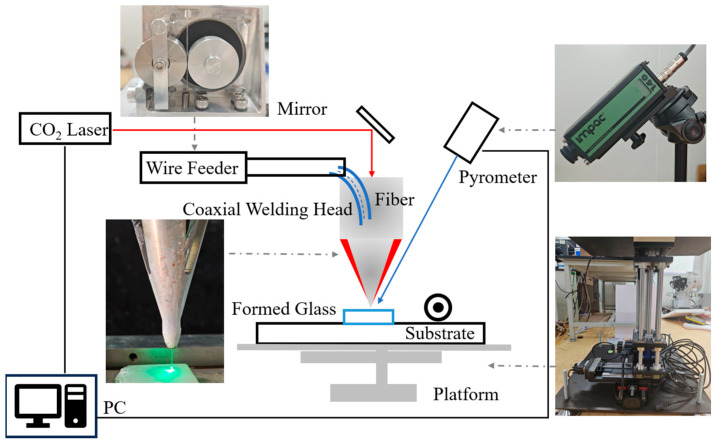
Schematic of quartz-glass laser additive manufacturing system.

A schematic of the experimental apparatus is shown in Figure 1. A computer controlled a Coherent 10.6 µm CO_2_ laser which had a 16 mm diameter Gaussian beam and a less than 2.0 mrad divergence angle. The beam passed through a 45° reflector and entered the laser head. In the laser head, the beam was first split and then focused by an off-axis parabolic mirror, forming a spot on the substrate mounted on a XYZ three-dimensional moving platform. At the same time, quartz-glass fiber passed through a wire feeder and the laser head, and the movement direction was perpendicular to the platform. The fiber absorbed energy at the laser spot, where it was melted and deposited on the substrate [15]. A pyrometer was installed in a side-axis manner to transmit the temperature of the glass melting area to the computer in real time. The substrate material in all experiments was pure quartz. It was 110 mm long, 25 mm wide, 6 mm thick, and the surface was polished. The diameter of the quartz-glass fiber was 0.6 mm, and its SiO_2_ mass fraction was greater than 99%.

Quartz glass is amorphous with no fixed melting point. Its softening temperature is approximately 1576 °C (1800 K), and its boiling point is 2226 °C (2500 K) [16,17]. Its visible and near-infrared band emissivity is low; it usually exhibits extremely high transparency and low absorption. Its emissivity in the mid-infrared range is higher, approaching 0.9 [18]. Therefore, an Impac IN 140/5-L (LumaSense, Germany) monochromatic pyrometer was used for temperature measurements over the range of 500–2500 °C and a temperature measurement band of 5.14 μm. The use of a monochromatic pyrometer requires that the object being measured fills the entire field of view. The minimum spot diameter of the CO_2_ laser beam simulated by ZEMAX 2019 was approximately 2 mm; hence, the minimum spot size of the variable focus lens for the pyrometer was 0.9 mm. In the wavelength range of 4.7–7.5 μm, quartz-glass emissivity was not temperature-dependent [19]; thus, the glass piece was placed in a 1000 °C furnace where its emissivity was calibrated to 93.6%.

The various process parameters are listed in Table 1. According to previous experiments, the defocus amount was set to 0, the experimental wire feeding speed was 5 mm/s, and the platform moving speed was 1 mm/s. Under this set of process parameters, the energy input in the additive process was appropriate, and quartz glass with good quality could be added more stably. Visual Studio 2022 was developed to integrate various devices for unified control and automation. After the experiments, the Leyes Z01-5-USB (Leyes Technology, China) digital microscope was used to magnify and observe the quartz glass produced by additive manufacturing. The Phenom XL scanning electron microscope (SEM, Thermo Fisher, Waltham, MA, USA) was used to observe its microscopic morphology with an accelerating voltage of 10 kV, and the pores in the SEM image were analyzed using Image-Pro Plus 6.0 software.

### 2.2. Control Design

The basic working mechanism of the closed-loop temperature control system constructed in the experiment is to use feedback control to adjust the temperature of the controlled object. First, a suitable target value is set as the temperature that the system expects to reach and maintain. The pyrometer monitors the temperature of the quartz glass during the additive process and transmits the measured value to the controller. The controller decides how to adjust the laser power based on the difference between the set value and the measured value. The controller has different algorithms to handle errors, and the PID control [20] widely used in industry was used here. The controller sends the adjustment command to the laser to adjust the laser power output so that the temperature of the system gradually approaches the set value. The pyrometer then continues to monitor and feedback new temperature data, and the controller recalculates and adjusts based on the updated error value.

**Figure 2:**
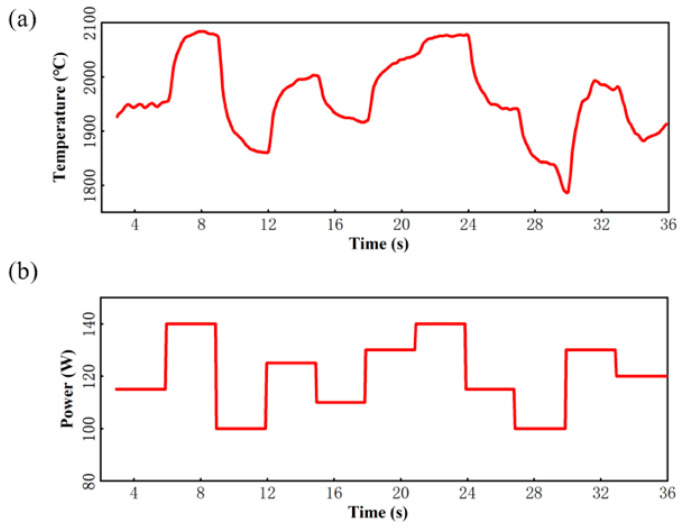
(**a**) Laser power input to the quartz-glass additive system. (**b**) Melting zone temperature corresponding to the laser power.

Because the physical aspects of laser additive manufacturing are complex, system modeling was performed by system identification [20,21,22,23]. The laser power was used as the control variable, and the state equation of the temperature control system was derived from experimental data. Because excessive laser power caused the quartz to evaporate or gasify, while insufficient laser power would not have allowed it to melt, the upper and lower limits of the laser power were set to 150 W and 100 W, respectively. The temperature sampling period was 0.05 s. According to the sampling theorem, the power signal pulse-width setting range was 3 s. Figure 2a is a randomly generated power signal, and Figure 2b is the temperature of the quartz-glass melting area in real time. Using the laser power data as input and the molten pool temperature data as output, a transfer function was obtained with the system identification toolbox; it is expressed by Equation (1).

(1) $$G(s)=(0.7088s^{3}+7.47s^{2}+179.9s+1.552)/(s^{3}+9.822s^{2}+11.23s+0.09973)$$

A PID control algorithm strategy was used, and a simulation model was established in Simulink according to Equation (1). Because the molten pool temperature had a significant impact on the optical and mechanical aspects of the additive quartz glass, the parameter adjustment goal was to reduce the adjustment time and overshoot as much as possible to rapidly stabilize the temperature. To facilitate the computer implementation of the model via conversion into a differential equation, the temperature control cycle was set to 0.2 s. After multiple adjustments via simulations and experiments, the obtained PID parameters in the control model were Kp = 0.3, Ki = 0.0144, and Kd = 0.05, where Kp is proportional gain, Ki is integral gain, and Kd is derivative gain.

## 3. Results and Discussion

### 3.1. Single-Channel, Single-Layer Experiment

#### 3.1.1. Open-Loop Experiment

**Figure 3:**
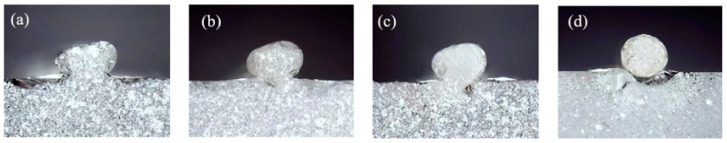
Cross-sectional morphologies of quartz glass produced with different laser powers: (**a**) 110 W, (**b**) 120 W, (**c**) 130 W, (**d**) 140 W.

Figure 3a–d show cross-sectional morphologies of quartz glass produced by additive manufacturing using laser powers of 110 W, 120 W, 130 W, and 140 W, respectively. The melting state and cross-sectional morphology changed significantly with laser power. For a 110 W power, Figure 3a shows a glass cross-section with an irregular-shaped surface and rough edges, indicating that the laser power did not adequately match the wire feeding speed. The rapid feeding of the wire material affected the full strength of the glass. The melts formed dents, resulting in poor molding results. As the power increased, the cross-sectional shape became more regular, the melting state became more uniform, and the glass surface became smoother. The cross-section for the 140 W power condition (Figure 3d) exhibited a complete structure with a glass sphere, indicating that the higher power provided sufficient thermal energy for melting and solidification. However, at the same time, the higher power caused the glass fiber to melt prematurely, and when it came into contact with the substrate, the cooling rates of the two were inconsistent, resulting in insufficient interface bonding.

**Figure 4:**
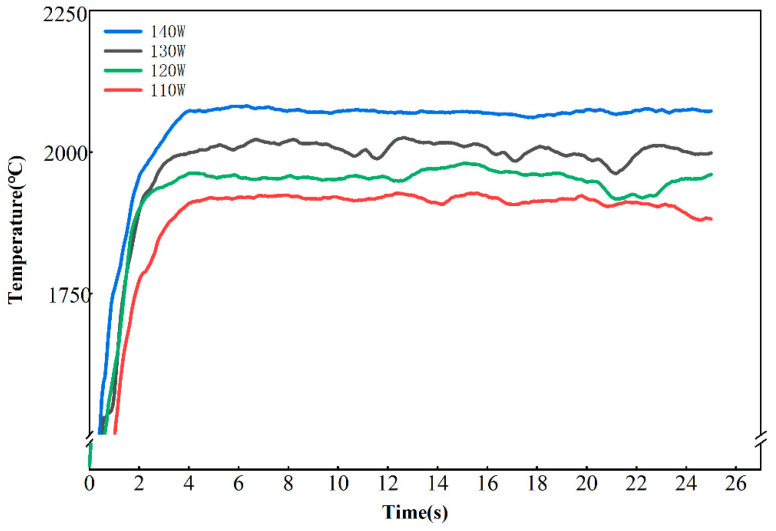
Temperature changes in the melting area at different constant powers.

In Figure 4, the temperatures during the additive processes at different laser powers are plotted. At each power, the temperature initially increased rapidly and then stabilized, indicating that the temperature control entered an equilibrium state. The stable temperature varied significantly with different power levels, reflecting the thermal equilibrium temperature of the material under different heating conditions.

As shown in Figure 3 and Figure 4, laser power played a key role in the flatness and structural integrity of the quartz-glass surface during additive manufacturing. When other process parameters remain unchanged (wire feeding speed was 5 mm/s, platform moving speed was 1 mm/s, and defocus was 0), because of the significant positive correlation between power (110–140 W) and temperature, the temperature could be controlled within a certain range to obtain the required processing conditions. This control could improve the macroscopic morphologies of additive structures and optimize the qualities of additive parts to meet requirements for various applications.

#### 3.1.2. Temperature Closed-Loop Experiment

**Figure 5:**
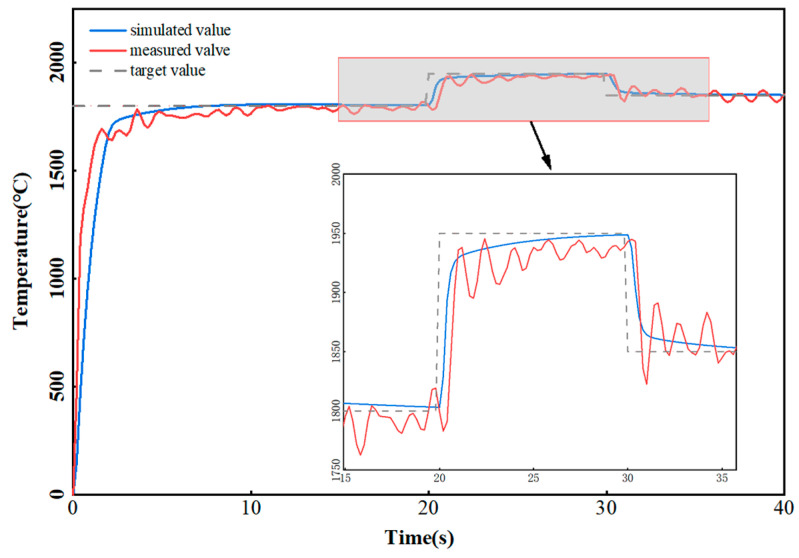
Temperature closed-loop control simulation and measurement.

In the single-channel, single-layer experiment, the preset temperature was initially 1800 °C, the expected temperature was set to 1950 °C at 20 s, and then the expected temperature jumped to 1850 °C at 35 s. Figure 5 plots the temperature setpoint, the closed-loop simulation results, and the actual temperature data. The modulation of the simulated temperatures and the actual values were consistent, indicating that the temperature could be quickly controlled around a set value. The actual temperature (red curve) increased rapidly in the initial stage, but then exhibited fluctuations, especially when approaching the set temperature, whereas the simulated value (blue curve) exhibited a smoother response without fluctuations and reached the target temperature more quickly. The fluctuation of the actual temperature was attributed to both the temperature instability of the melting zone and pyrometer uncertainty and repeatability. The uncertainty was 1.2% of the reading and the repeatability was 0.3% of the reading. The measurement error during the additive process could reach 30 °C.

### 3.2. Single-Layer Multi-Channel Experiment

In the single-track, single-layer open-loop experiment (Figure 4), the temperature was stable during additive manufacturing, even without temperature control, provided that a constant laser power was applied. However, in practical applications, additive manufacturing is often used to print three-dimensional structures. Therefore, open-loop and closed-loop experiments for multi-track, single-layer additive manufacturing were compared and analyzed to investigate the effect of temperature control on the fabrication of complex structures.

#### 3.2.1. Macroscopic Morphology

**Figure 6:**
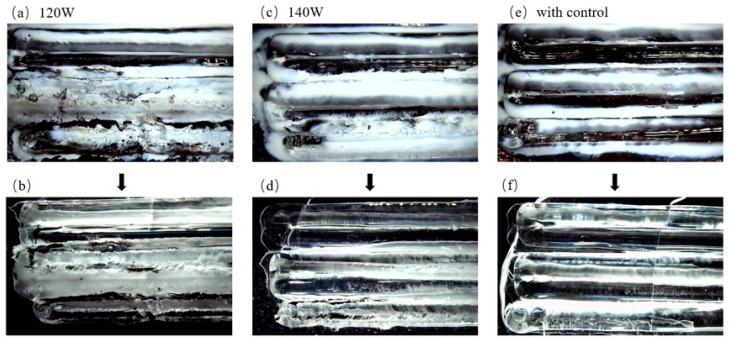
Macroscopic morphologies of quartz under different additive conditions: (**a**) 120 W, before pickling; (**b**) 120 W, after pickling; (**c**) 140 W, before pickling; (**d**) 140 W, after pickling; (**e**) after temperature control, before pickling; (**f**) after temperature control, after pickling.

**Figure 7:**
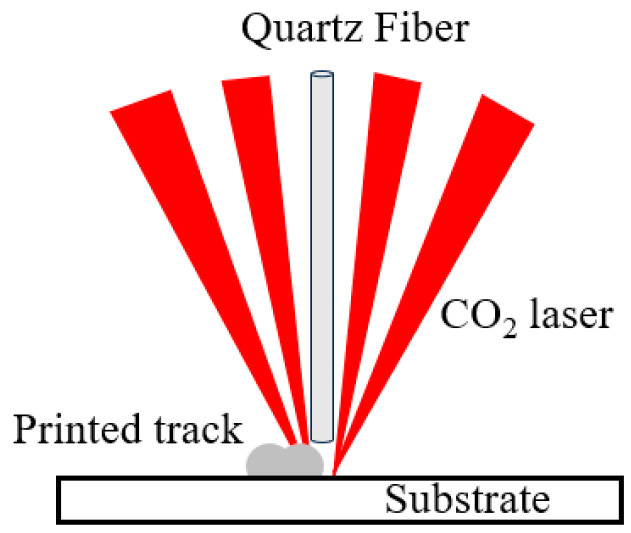
The laser can be blocked by the added area.

A multi-pass single-layer additive experiment was conducted on a quartz optical fiber, with the spacing between each pass set to 1.2 mm, with constant powers of 120 W and 140 W, and with other parameters left unchanged. The results were compared with those of closed-loop temperature control. Each image in Figure 6 is the result of the first to fifth additive passes from top to bottom. It shows the experimental results for the 1.2 mm spacing, where Figure 6a,c,e show the direct additive effect, and Figure 6b,d,f show the additive results after pickling in low-concentration hydrofluoric acid was used to wash away glass crystals that precipitated during the additive process. Because the CO_2_ laser was initially split and then focused, and if the interval between each additive pass was short, then, starting from the second pass, part of the additive in the front blocked part of the laser, resulting in a reduction in the energy at the additive position. This effect is depicted in Figure 7 and will accumulate with the number of additives, reducing the quality and, finally, causing the optical fiber to break and stop additives. Therefore, the 140 W constant power exhibited quality defects at a later time than did the constant power of 120 W. For example, defects began to appear in the fourth pass at 140 W (Figure 6c), while defects appeared in the second pass at 120 W (Figure 6a). However, the surfaces of the quartz additives after temperature closed-loop control were relatively smooth, transparent, and clear, and the macroscopic morphologies were basically the same.

#### 3.2.2. Microstructure

**Figure 8:**
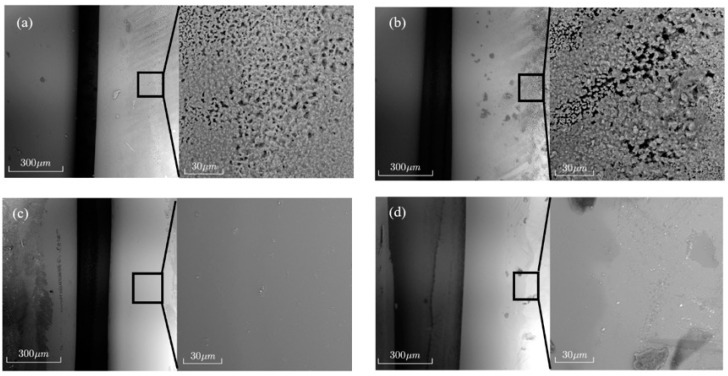
Quartz microstructures under different additive conditions: (**a**) interval of 1.2 mm, 120 W; (**b**) interval of 1.2 mm, 140 W; (**c**) interval of 1.2 mm, after temperature control; (**d**) interval of 1.5 mm, 140 W.

A constant power of 140 W and an interval of 1.5 mm were used to supplement the multi-pass single-layer experiment. Together with the quartz glass obtained from the above experiments, a layer of gold powder was plated on the surface via ion magnetron sputtering to enhance the scanning electron microscopy (SEM) imaging of microstructures in random surface areas of the second additive. Figure 8a–c show SEM images of sample surfaces magnified 285 times and 2000 times compared to those in Figure 6b,d,f. Figure 8d is an SEM image of the surface magnified 285 times and 2000 times after the sample was acid-washed following a constant power of 140 W and an interval of 1.5 mm.

Figure 8a–c show microstructural comparisons of the surface of additively manufactured quartz glass before and after temperature control. Before temperature control (Figure 8a,b), there was pore distribution and significant roughness on the surface, indicating uneven cooling or melting during material deposition. A high gas retention rate could have generated structural defects, which could affect light transmission and mechanical properties and reduce high-precision applications. With temperature control (Figure 8c), the surface was smoother, the structure was flatter and denser, and the number of pores was significantly reduced. Temperature control optimization could improve the melting and cooling of the material, thereby reducing pore formation and microscopic defects, making the additively manufactured quartz glass more stable in terms of physical and chemical properties. This improved the structural integrity of the material and its optical and mechanical properties. When the power was constant at 140 W with a 1.5 mm additive interval, and only these process parameters were changed, the surface was relatively smooth (Figure 8d), with no bubbles and a small number of attached impurities. The surface quality was close to the results using temperature control.

**Figure 9:**
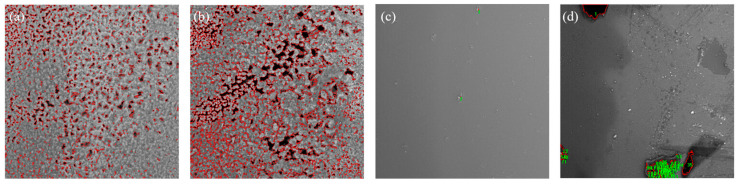
Quartz surface porosity under different additive conditions: (**a**) 9.793% porosity, interval of 1.2 mm, 120 W; (**b**) 19.723% porosity, interval of 1.2 mm, 140 W; (**c**) 0.101% porosity, interval of 1.2 mm, after temperature control; (**d**) 1.656% porosity, interval of 1.5 mm, 140 W.

Image-Pro Plus software was used to perform porosity analysis on the scanning electron microscope (SEM) images, and the results are shown in Figure 9 (a–d in Figure 9 correspond to a–d in Figure 8, respectively). During analysis, pore areas were highlighted in red. It was calculated that the porosity is 9.793% in Figure 9a, and the pores are mainly uniformly distributed in the sample in terms of small sizes and large numbers. The porosity is 19.723% in Figure 9b, the pore distribution has high connectivity, and the pore size is larger than that of the sample in Figure 9a. With a porosity of 0.101% in Figure 9c, the porosity is so low that the sample could almost be considered a dense material, and the impact of pores in its microstructure on properties is negligible. The porosity is 1.656% in Figure 9d; the pores are sparsely distributed, the number is significantly reduced, and the density of the material is significantly improved.

This indicated that appropriate process parameter design was the key to ensuring manufacturing quality. However, temperature control was still important during additive manufacturing to further optimize the process under specific conditions.

## 4. Conclusions

A closed-loop temperature controller based on PID control was designed and tested. It rapidly and accurately adjusted the output power of a CO_2_ laser to change the deposition state of the quartz-glass melting area. During the laser additive manufacturing of quartz glass, excessive or insufficient energy input may occur because of improper process parameters or interference from external factors, such as the optical fiber and laser not being completely coaxial and fluctuations in wire feed rates. With a closed-loop temperature controller, the system could always maintain a stable thermal equilibrium state during the additive process. This significantly reduced bubbles in the additive sample, improved surface smoothness and structural density, and ensured quality consistency. This method of laser additive manufacturing effectively optimized the surface morphology and material properties of the quartz glass, improved the yield, and provided a foundation for the future rapid additive manufacturing of quartz glass requiring specific morphologies or higher additive quality.

## Figures and Tables

**Table 1:** Process parameters for quartz-glass additive experiments.

Process Parameters	Numeric
Laser power	100–150 W
Wire feeding speed	5 mm/s
Platform moving speed	1 mm/s
Defocus	0

## Data Availability

The original data presented in this study are included in the article. Further inquiries can be directed to the corresponding author.
